# A rare mechanism of delayed splenic rupture following the nonoperative management of blunt splenic injury in a child

**DOI:** 10.1186/s40792-018-0477-5

**Published:** 2018-07-11

**Authors:** Toko Shinkai, Kentaro Ono, Kouji Masumoto, Yasuhisa Urita, Chikashi Gotoh

**Affiliations:** Department of Pediatric Surgery, Faculty of Medicine, University of Tsukuba, 1-1-1, Tennoudai, Tsukuba, Ibaraki 305-8575 Japan

**Keywords:** Blunt splenic injury, Nonoperative management, Delayed splenic rupture, Pediatric

## Abstract

**Background:**

Nonoperative management (NOM) has been established as the standard treatment for isolated blunt organ injury in hemodynamically stable pediatric patients. Although delayed splenic rupture or bleeding is a rare complication in NOM, it is an issue that many pediatric surgeons are greatly concerned about. We herein report a rare pediatric case concerning the mechanisms involved in delayed splenic rupture after NOM.

**Case presentation:**

A 9-year-old boy with severe abdominal pain was transferred to our hospital. Twenty-one hours before the admission, he had been kicked in the region of his left lateral abdomen. Contrast-enhanced abdominal computed tomography revealed a severe intra-parenchymal hematoma and multiple lacerations of the spleen with a large amount of hemoperitoneum without active bleeding. His condition was diagnosed as a grade III injury on the AAST splenic injury scale. After fluid resuscitation, his vital signs became stable. The patient was treated with NOM in our intensive care unit. However, suddenly after defecation (72 h after the injury), he started complaining of severe abdominal pain and left shoulder pain. His blood pressure dropped to 70/35 mmHg, and he started to lose consciousness. Abdominal ultrasonography (US) revealed increased ascites. Fluid resuscitation and blood transfusion were performed. His symptoms and abdominal US findings suggested that splenic re-bleeding had caused delayed splenic rupture to occur. Emergency splenectomy was performed. The resected spleen was enlarged with a large parenchymal hematoma. The posterior-lateral side of the splenic capsule was ruptured.

**Conclusions:**

The mechanism of delayed splenic rupture in our case was considered to be the result of a tear in the subcapsular hematoma caused by stretching the splenocolic ligament related to a bowel movement during defecation. Although delayed splenic rupture or bleeding is unpredictable, it is very important to understand the mechanisms and to educate the family of the children with splenic injuries of the warning signs of delayed rupture or bleeding.

## Background

Among blunt abdominal injuries in children, the spleen is the most frequently injured organ [1]. Nonoperative management (NOM) has been established as the standard treatment for isolated blunt organ injuries in hemodynamically stable pediatric patients [2, 3]. The high success rate of NOM in isolated blunt splenic injuries has been reported [4–6]. Although delayed splenic rupture or bleeding is a rare complication [4–7], it is a potential problem that requires careful observation, both during hospital stays and after discharge [3, 7].

We herein report on a case of delayed splenic rupture caused by tearing of a subcapsular hematoma. We believe the mechanism causing the splenic capsule to tear involved stretching the splenocolic ligament, leading to colonic peristalsis during defecation.

## Case presentation

A 9-year-old boy (height: 133 cm, weight: 25.8 kg) with severe abdominal pain was transferred to our hospital. Twenty-one hours before admission, he had been kicked in the region of his left lateral abdomen. On admission, the patient’s face was pale, and his Glasgow coma scale was recorded at 13/15 (E: 3, V: 4, M: 6). A physical examination revealed severe tenderness on the left lateral abdomen with moderate abdominal distension. His vital signs were as follows: blood pressure 80/50 mmHg, heart rate 110 bpm, and a respiratory rate of 30/min, respectively.

**Fig. 1:**
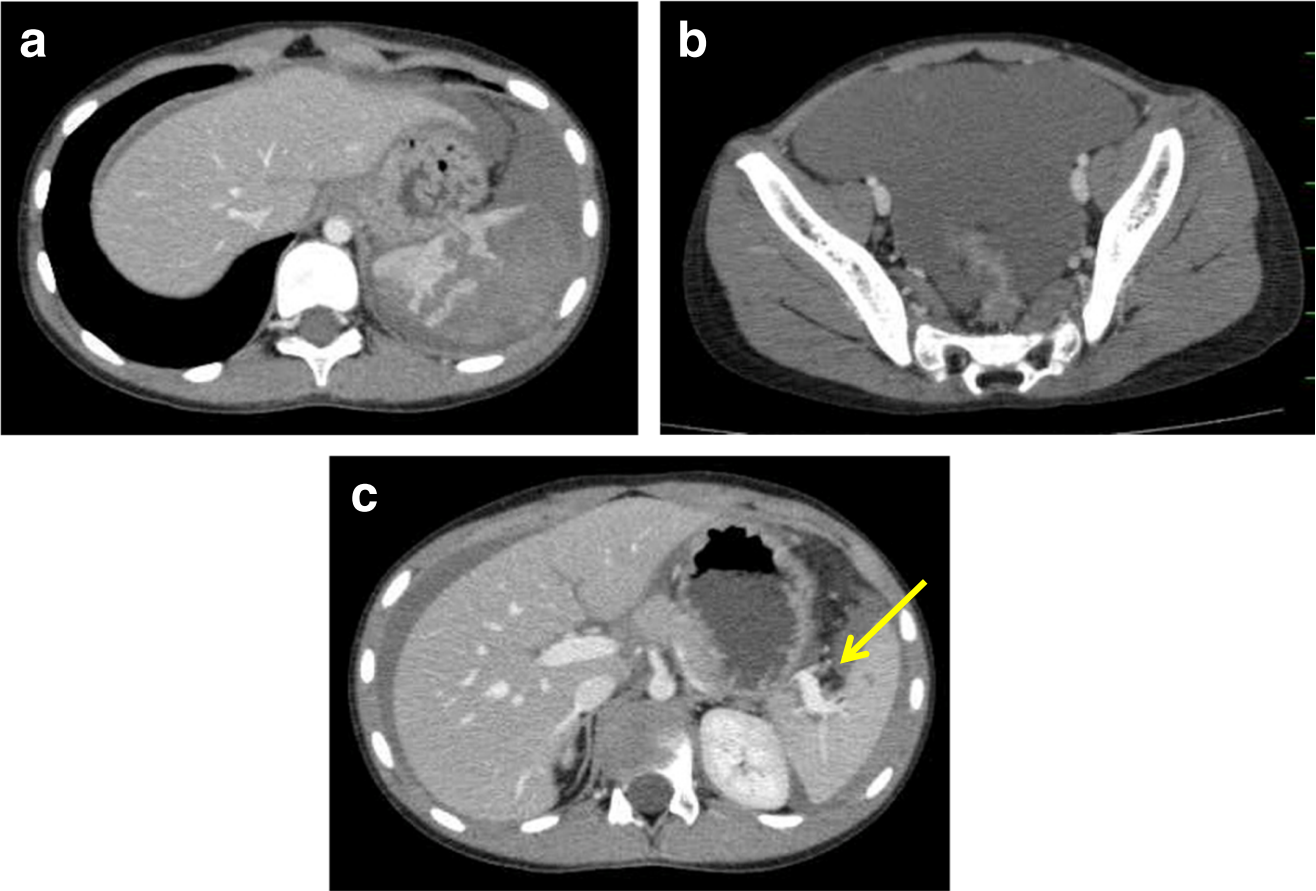
Contrast-enhanced CT at admission (21 h after injury). **a** Severe intraparenchymal hematoma and multiple lacerations of the spleen were noted. **b**. A large amount of hemoperitoneum was detected in the pelvic cavity. **c** The hilar vessels of the spleen were not injured, and no active bleeding was seen in the spleen (→). The injury was diagnosed as AAST grade III

Contrast-enhanced abdominal computed tomography revealed a severe intraparenchymal hematoma and multiple lacerations of the spleen with a large amount of hemoperitoneum (Fig. 1a, b). The splenic hilar vessels were not injured (Fig. 1c), and there were no signs of active bleeding. His injury was diagnosed as grade III on the American Association for the Surgery of Trauma (AAST) splenic injury scale. The patient’s laboratory data showed his red blood cell count 251 × 10^4^/ml, hemoglobin (Hb) 9.1 g/dl, hematocrit (Ht) 27.1%, and platelets 33.6 × 10^4^/μl. All of his other laboratory data were within normal limits. After fluid resuscitation, his blood Hb and Ht levels fell to 7.6 g/dl and 22.3%, respectively. He required a transfusion of 1000 ml red blood cells in order to maintain his blood pressure. His vital signs became stable, and he regained clear consciousness.

**Fig. 2:**
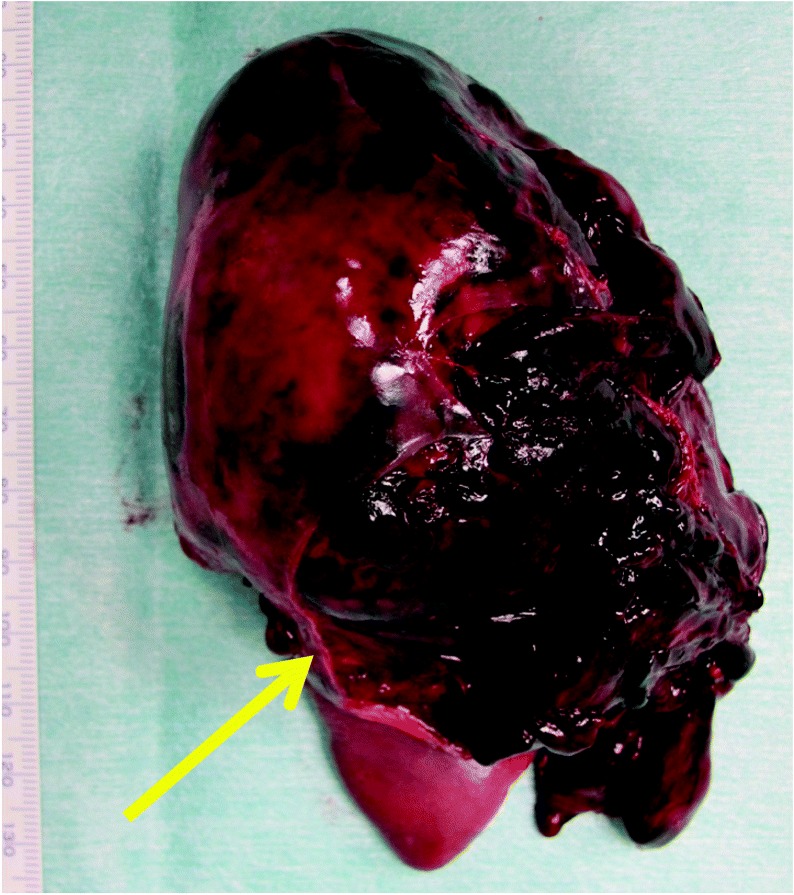
Gross findings of the resected spleen. The resected spleen was enlarged with a large parenchymal hematoma. The postero-lateral side of the splenic capsule was torn (→)

We treated him by NOM in our intensive care unit. His abdominal pain decreased gradually while resting in bed. His hemodynamic conditions were stable, and further blood transfusion was not necessary. At 70 h after the injury, he started drinking water. Two hours after drinking water (72 h post-injury), he discharged feces. After defecation, he suddenly started complaining of severe abdominal pain and left shoulder pain. His blood pressure dropped to 70/35 mmHg, and he started to lose consciousness. Abdominal ultrasonography (US) revealed increased ascites. Fluid resuscitation and blood transfusion were performed. His symptoms and abdominal US findings suggested that splenic re-bleeding had caused delayed splenic rupture to occur. An emergency splenectomy was performed. The resected spleen was enlarged with a large parenchymal hematoma. The posterior-lateral side of the splenic capsule was torn (Fig. 2). The splenocolic ligament was resected during the operation. There were no anatomical anomalies between the ligament and the spleen.

The post-operative course was uneventful, and the patient was discharged 9 days after splenectomy.

## Discussion

The standard procedure for managing isolated blunt splenic injuries for hemodynamically stable pediatric patients is NOM [2, 3]. The success rate of NOM has been reported at 90–100% [4–6]. However, delayed complications have been reported, including pseudocysts, abscess, pseudoaneurysms, and delayed rupture [1, 8]. Among these complications, delayed splenic bleeding is the most severe. Delayed splenic bleeding is an extremely rare complication in children, with incidence rates of only 0 to 0.33% [4–7]. However, delayed splenic rupture or bleeding is very serious due to its high mortality rate, reported at 15% in adult cases [9]. Various theories concerning the cause of delayed splenic bleeding include tears in a subcapsular hematoma, clot disruption, rupture of pseudoaneurysm, and internal splenic pseudocyst bleeding [7, 9].

Rupture typically occurs 48 to 72 h after injuries in cases diagnosed as delayed splenic rupture [10]. However, Jen et al. [4] described four cases of delayed splenic rupture that occurred 4 to 20 days post-injury. Davis et al. [7] reviewed cases of delayed splenic bleeding and found that they occurred 2 to 28 days post-injury. In our case, splenic rupture occurred 72 h post-injury, during the patient’s hospital stay. Some cases occur after discharge from the hospital. It is very important to educate the family of a child with a splenic injury about the warning signs of delayed rupture or bleeding [3, 7]. Patients with symptoms of increased pain, pallor, dizziness, vomiting, and worsening shoulder pain must be returned to the hospital [3].

Treatment options of delayed splenic rupture and bleeding include splenectomy, splenorrhaphy, splenic artery embolization (SAE), and observation. Twenty cases of delayed splenic rupture and bleeding were reported [4, 5, 7, 8]. Eighteen patients underwent splenectomy or splenorrhaphy. SAE was performed in one case and observation in the other. Although we performed emergency splenectomy, SAE might be considered for hemodynamically stable patients to preserve the splenic function so as to avoid postsplenectomy sepsis.

The mechanism of delayed splenic rupture in our case was considered to be the result of a tear in the subcapsular hematoma caused by stretching of the splenocolic ligament. The patient started complaining about abdominal and shoulder pain suddenly after defecation, which led us to suspect that the bowel movement had caused the splenocolic ligament to stretch at the splenic flexure. Also, defecation might cause rising of intra-abdominal pressure that could induce a rupture of the hematoma. There have also been a few reports of splenic rupture as a complication of colonoscopy [11]. One of the reported mechanisms underlying such complications is stretching of the splenocolic ligament during colonoscopy, which can lead to avulsion or tearing of the spleen. This mechanism of delayed splenic rupture is considered to be rare and unpredictable. However, other reports describe cases of delayed splenic rupture caused by laceration of a perisplenic hematoma stretched by distending the stomach after eating a heavy meal [12].

Given our understanding of the mechanisms involved in delayed splenic rupture, we strongly recommend careful observation of patients after beginning oral intake. It is very important to understand that gastrointestinal peristalsis can cause delayed splenic rupture.

## Conclusions

We herein report a rare case of delayed splenic rupture in a pediatric patient undergoing NOM of blunt splenic injury. Stretching of the splenocolic ligament during bowel peristalsis can cause tears in a subcapsular hematoma. It is very important to provide information to the family regarding the symptoms and signs of delayed splenic rupture or bleeding.

## Abbreviations

AAST: American Association for the Surgery of Trauma
CT: Computed tomography
NOM: Nonoperative management
US: Ultrasonography
